# Mortality Rate and Cause of Death in Adults with Extrapulmonary Nontuberculous Mycobacteria Infection, Denmark

**DOI:** 10.3201/eid3009.240475

**Published:** 2024-09

**Authors:** Andreas A. Pedersen, Victor N. Dahl, Anders Løkke, Inge K. Holden, Andreas Fløe, Rikke Ibsen, Ole Hilberg, Isik S. Johansen

**Affiliations:** University of Southern Denmark, Odense, Denmark (A.A. Pedersen, A. Løkke, I.K. Holden, O. Hilberg, I.S. Johansen);; Lillebaelt Hospital, Vejle, Denmark (A.A. Pedersen, A. Løkke, R. Ibsen, O. Hilberg);; Mycobacterial Centre for Research Southern Denmark, Odense (A.A. Pedersen, I.K. Holden, O. Hilberg, I.S. Johansen);; Odense University Hospital, Odense, Denmark (A.A. Pedersen, I.K. Holden, I.S. Johansen);; Aarhus University Hospital, Aarhus, Denmark (V.N. Dahl, A. Fløe)

**Keywords:** tuberculosis and other mycobacteria, mycobacteria, nontuberculous mycobacterial disease, extrapulmonary nontuberculous mycobacterial disease, epidemiology, mortality, causes of death, Denmark

## Abstract

Evidence on mortality rates and causes of death associated with extrapulmonary nontuberculous mycobacteria (NTM) infection is limited. This nationwide register-based study in Denmark used diagnostic codes to match adult patients with extrapulmonary NTM infection 1:4 to controls. During 2000–2017, we identified 485 patients, who had significantly more comorbidities than controls. The 5-year mortality rate for patients was 26.8% (95% CI 23.1%–31.0%) and for controls, 10.9% (95% CI 9.6%–12.4%). The median age at death was 76 (interquartile range 63–85) years for patients and 84 (interquartile range 73–90) years for controls. The adjusted hazard rate of death for patients was 1.34 (95% CI 1.10–1.63; p = 0.004). Patients and controls mainly died of cardiovascular disease and solid malignant neoplasms. Hematologic malignancies and HIV were more frequently causes of death in patients. Mortality rates are substantial among patients with extrapulmonary NTM infection, predominantly caused by underlying conditions.

Extrapulmonary nontuberculous mycobacteria (NTM) infection potentially affects any organ; lymph nodes, skin, and soft tissue are the most commonly affected (*1**,**2*). The severity of disease varies considerably from uncomplicated lymphadenitis with favorable prognosis among children to disseminated disease in immunocompromised patients (*3**–**5*). Still, a substantial knowledge gap remains regarding outcomes of extrapulmonary NTM disease (*6*).

Historically associated with HIV and AIDS, incidence of extrapulmonary NTM infection has been increasing and represents a growing healthcare challenge (*1**–**3**,**7*). This increase has been attributed to several factors, such as the intensified use of immunosuppressants, increased life expectancy, and increased awareness of the disease (*1**,**3**,**8**,**9*). Furthermore, extrapulmonary NTM has been implicated in iatrogenic infections and healthcare-related outbreaks (*10**–**13*). In addition, the discontinuation of the bacillus Calmette-Guérin (BCG) vaccine in areas with low tuberculosis incidence might have contributed to this increase, possibly because of reduced nonspecific immune protection previously provided by the vaccine (*14*)

Studies on extrapulmonary NTM–related deaths are scarce, and reported mortality rates vary widely. In a US cohort of 365 NTM infections, the mortality rate for extrapulmonary NTM was 2% and for disseminated NTM disease was 50% (*15*). In contrast, another US study of 831 extrapulmonary NTM patients from 2009–2014 reported an overall crude mortality rate of 5% (11% disseminated disease and 2% skin and soft tissue infections) (*16*). In patients with hematologic cancers, 30-day mortality has been reported at 15% in NTM-infected persons, compared with only 2% in noninfected persons (*17*).

In summary, extrapulmonary NTM represents a highly heterogeneous disease entity in terms of clinical manifestation and host factors, and knowledge of its associated mortality rates and causes of death among persons with extrapulmonary NTM disease is limited. To address this knowledge gap, we investigated mortality rates associated with diagnosis of extrapulmonary NTM infection and describe the causes of death.

## Methods

### Study Design

This study was a nationwide retrospective register-based cohort study from Denmark. The healthcare system is based on tax-funded universal healthcare. Different types of registers can be linked by using a unique 10-digit personal identification number issued to all citizens (*18*).

### Data Sources and Measurement

The Danish Register of Causes of Death contains data on time, place, and cause of death using the International Classification of Diseases, 10th Revision (ICD-10), for all deaths occurring in Denmark (*19*). We identified all patients with extrapulmonary NTM infection using International Classification of Diseases, 8th Revision (ICD-8), and ICD-10 diagnostic codes from the National Patient Register. The register contains the ICD-8 and ICD-10 diagnostic codes and procedural codes on all inpatients from 1977 and all outpatients since 1995 (*20*). To evaluate the effects of baseline comorbidities on mortality, we calculated a Charlson Comorbidity Index (CCI) as described by Quan et al. (*21*) using ICD-10 codes from the National Patient Register.

### Study Subjects

We included patients >18 years of age with a first-time extrapulmonary NTM disease ICD-10 code (A31.1, A31.8, or A31.9) during 2000–2017. The date of the first registered extrapulmonary NTM code was considered the index date. For persons without an extrapulmonary NTM–specific ICD-10 code (A31.1), we used procedural codes for specific examinations and treatments to differentiate between extrapulmonary NTM and other NTM disease manifestations in windows of 1 year on either side of the index date (Appendix Table). Patients and controls were censored from the study at death, migration, or end of data. We matched patients with extrapulmonary NTM disease to controls at a ratio of 1:4 at index. Controls were randomly selected from the total population of Denmark and matched by birth year, sex, marital status, and municipality of residence. The controls entered the study at the same date as the case-patient to whom they were matched. We excluded persons with an ICD-8 or ICD-10 code of A31 from the possible control population to ensure a nonbiased representative sample.

### Statistical Analysis and Variables

We present absolute numbers and percentages or medians with interquartile ranges (IQRs) as appropriate. We evaluated differences between groups using the χ^2^ test and median age differences using the Wilcoxon-Mann-Whitney nonparametric test. Because of legislation in Denmark, descriptive variables with <3 observations were not reported to ensure patient privacy. We evaluated mortality rates using a cumulative mortality plot and tested differences using a log-rank test. We used the Cox proportional hazards model to estimate unadjusted hazard ratios (HRs) of death and adjusted for CCI (*21*). Because HRs were not constant over time (nonproportional), we estimated annual and average HRs in the study period (*22*). To investigate the effects of HIV and AIDS on mortality, we performed a sensitivity analysis by excluding patients with HIV from the analyses. We post hoc categorized comorbidities into 3 groups (overall burden of comorbidities, break in barrier function, and impaired immunity) on the basis of existing knowledge of risk factors for NTM infection to investigate the association of comparable comorbidities and morbidity (*9*,*10*,*12*,*23*–*25*). We examined causes of death using the 21 World Health Organization ICD-10 classification groups for cases and controls and qualified them using detailed data on causes of death for the most common groups for extrapulmonary NTM compared with controls. We performed statistical analyses using Stata version 16.1 (StataCorp LLC, https://www.stata.com) and SAS version 9.4 TS Level 1M5 (SAS Institute Inc., https://www.sas.com). We applied a significance level of 0.05 for all tests.

### Ethics Statement

Ethics approval is not required for register studies according to Danish law. The Region of Southern Denmark (jr. no. 22/10240) approved the study.

## Results

### Patient Demographics

For the period spanning 2000–2017, we identified 485 patients with extrapulmonary NTM disease. Their median age at index was 57 (IQR 41–73) years; 40.4% of patients were women and 59.6% men, and 49.6% were married or cohabiting (Table 1). The median follow-up duration was 5 (IQR 3–10) years for extrapulmonary NTM patients and 6 (IQR 3–11) years for controls. NTM diagnostic codes for cutaneous infection accounted for 29.7% (*Mycobacterium marinum* in 13.2%, *M*. *ulcerans* in 1%), whereas 70.3% were reported as other or not otherwise specified.

**Table 1:** Characteristics of patients and controls in study of mortality rates and cause of death in adults with extrapulmonary NTM infection, Denmark*

Characteristic	Extrapulmonary NTM		Controls	
No. persons	485		1,935	
Sex			Matched	
F	196 (40.4)			
M	289 (59.6)			
Median age, y (IQR)	57 (41–73)		Matched	
Age group, y				
18–29	46 (9.5)			
30–39	61 (12.6)			
40–49	77 (15.9)			
50–59	81 (16.7)			
60–69	79 (16.3)			
>70	141 (29.1)			
Marital status			Matched	
Married/cohabiting	282 (49.6)			
Not married/cohabiting	203 (50.4)			
Immigration status†				
Non-Western origin	46 (9.5)		111 (5.7)	
Western/Danish origin	438 (90.3)		1,822 (94.2)	
NTM diagnostic codes				
Cutaneous mycobacterial infection NOS	75 (15.5)			
Cutaneous infection with *Mycobacterium marinum*	64 (13.2)			
Cutaneous infection with *Mycobacterium ulcerans*	5 (1.0)			
Other mycobacterial infections	73 (15.1)			
Mycobacterial infection NOS	268 (55.3)			
CCI				
Mean CCI (SD)	1 (1.83)		0.26 (0.81)	
CCI group				
0	313 (64.5)		1,694 (87.5)	
1	48 (9.9)		74 (3.8)	
2	58 (12.0)		127 (6.6)	
>3	66 (13.6)		40 (2.1)	

*Values are no. (%) except as indicated. CCI, Charlson Comorbidity Index; IQR, interquartile range; NOS, not otherwise specified; NTM, nontuberculous mycobacteria. †Unknown status for extrapulmonary NTM n = 1 and control n = 2.

### Mortality Rates

The median age at death from all causes was 76 (IQR 63–85) years for extrapulmonary NTM patients compared with 84 (IQR 73–90) years in controls. In total, 158 (32.6%) of 485 extrapulmonary NTM patients died during the study period. Extrapulmonary NTM patients had a significantly higher mortality rate than did matched controls (mortality HR 1.9, 95% CI 1.58–2.29; p<0.0001); the largest difference occurred in the first 3 years after diagnosis. The cumulative 1-year mortality rate for extrapulmonary NTM patients was 9.3% (95% CI 7.0%–12.2%), compared with 3.1% (95% CI 2.4%–4.0%) for controls. The cumulative 5-year mortality rate was 26.8% (95% CI 23.1%–31.0%) for extrapulmonary NTM patients and 10.9% (95% CI 9.6%–12.4%) for controls (Figure).

**Figure:**
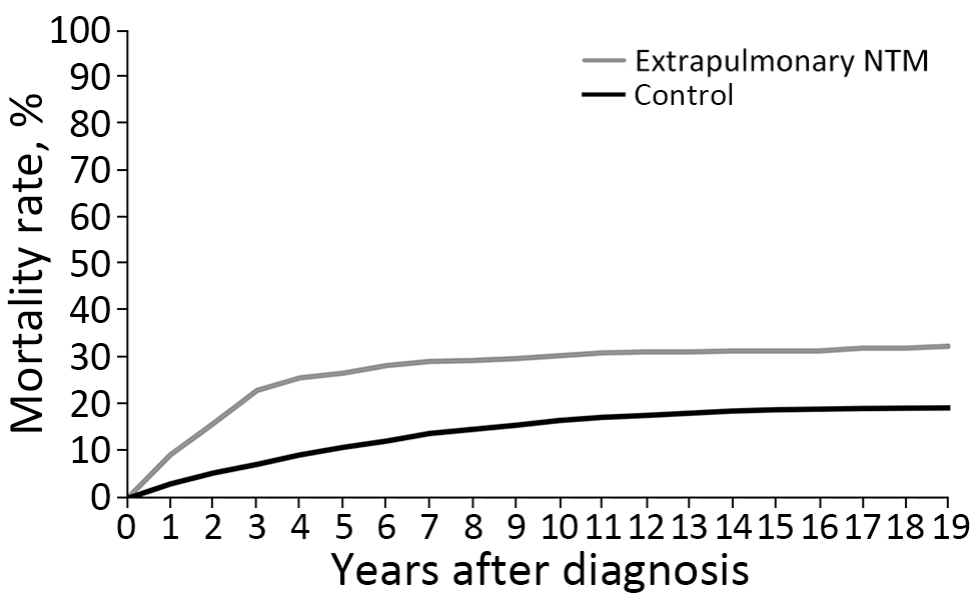
Cumulative mortality rate for patients with extrapulmonary NTM infection compared with matched controls in study of mortality and cause of death in adults with extrapulmonary NTM infection, Denmark. log-rank p<0.001. NTM, nontuberculous mycobacteria.

The increased mortality rate remained after adjusting for baseline CCI with an HR of 1.34 (95% CI 1.10–1.63; p<0.01). The effect was most pronounced in the first 3 years (Table 2). After 4 years of follow-up, the hazards of death did not differ between extrapulmonary NTM patients and controls. The hazards of death remained higher for extrapulmonary NTM patients even when excluding persons with HIV from the analysis (HR 1.4, 95% CI 1.2–1.7).

**Table 2:** Cox hazard regression for patients with extrapulmonary NTM infection compared with matched controls adjusted for Charlson Comorbidity Index and nonproportionality over time in study of mortality and cause of death in adults, Denmark*

Category	Extrapulmonary NTM versus controls		
	HR (95% CI)		p value
Total	1.34 (1.1–1.6)		0.004
Year after diagnosis			
1	2.07 (1.6–2.7)		
2	1.83 (1.3–2.5)		
3	1.61 (1.1–2.4)		
4	1.43 (0.9–2.2)		
5	1.26 (0.8–2.1)		
6	1.11 (0.6–1.9)		
7	0.98 (0.5–1.8)		
8	0.87 (0.4–1.7)		
9	0.77 (0.4–1.6)		
10	0.68 (0.3–1.5)		

*HR, hazard ratio for death; NTM, nontuberculosis mycobacteria.

### Causes of Death

Patients with extrapulmonary NTM disease died more frequently from infectious diseases (10.8%, 95% CI 6.4–16.7) than did controls (2.1%, 95% CI 0.1–4.2). Cardiovascular disease and solid malignant neoplasms were the 2 most common causes of death for extrapulmonary NTM patients and for controls (Table 3). Cardiovascular disease was the cause of death for 17.7% of extrapulmonary NTM patients and 24.0% of controls, whereas solid malignant neoplasms caused 13.9% of deaths for extrapulmonary NTM patients and 16.5% of deaths for controls. Deaths caused by hematologic malignancies were significantly more common among extrapulmonary NTM patients (7.6%, 95% CI 4.0%–12.9%) than among controls (3.5%, 95% CI 1.9%–5.9%; p<0.05). Other bacterial diseases, a grouping that potentially includes NTM disease, accounted for 5.7% (95% CI 2.6%–10.5%) of deaths but was not reported for controls.

**Table 3:** Causes of death for patients with extrapulmonary NTM infection and controls, Denmark*

Category	Extrapulmonary NTM			Controls		
	Cause of death	No. (%)		Cause of death	No. (%)	
All causes	Total	158 (32.6)		Total	375 (19.4)	
	Male	97 (33.6)		Male	256 (22.2)	
	Female	61 (31.1)		Female	119 (15.2)	
Rank						
1	Cardiovascular disease	28 (17.7)		Cardiovascular disease	90 (24.0)	
2	Solid malignant neoplasms	22 (13.9)		Solid malignant neoplasms	62 (16.5)	
3	Hematological malignancies	12 (7.6)		Organic mental disorders	18 (4.8)	
4	Other bacterial diseases†	9 (5.7)		Influenza and pneumonia	16 (4.3)	
5	Respiratory diseases	8 (5.1)		Respiratory diseases	13 (3.5)	
6	HIV	6 (3.8)		Hematological malignancies	13 (3.5)	
	Others	73 (46.2)		Others	163 (43.5)	

*NTM, nontuberculosis mycobacteria. †Codes A30–A49, including mycobacterial diseases, in International Classification of Diseases, 8th Revision or 10th Revision.

### Comorbidities

#### Overall Burden of Comorbidities

Persons with extrapulmonary NTM infection had higher CCI scores and an overall significantly higher burden of comorbidities than did controls (Table 1). In particular, the burden of cardiovascular diseases was significantly higher in extrapulmonary NTM patients at 38.8% (95% CI 34.4%–43.3%) than among controls at 19.3% (95% CI 17.6%–21.2%). The burden of chronic lower respiratory diseases was also significantly higher in extrapulmonary NTM patients at 6.2% (95% CI 4.2%–8.7%) than among controls at 2.5% (95% CI 1.9%–3.3%) (Table 4). Metabolic disorders, benign neoplasms, diseases of the male genitalia, and diseases of the gastrointestinal tract were also more common in extrapulmonary NTM patients.

**Table 4:** Numbers and percentages of comorbidities 3 years before diagnosis in study of mortality and cause of death in adults with extrapulmonary NTM infection, Denmark*

Comorbidity	Patients with extrapulmonary NTM			Controls	
	No.	% (95% CI)		No.	% (95% CI)
Cardiovascular disease	188†	38.8 (34.4–43.3)		373	19.3 (17.6–21.2)
Head and musculoskeletal injuries	142†	29.3 (25.3–33.6)		330	17.1 (15.4–18.8)
Other bacterial diseases including pneumonia‡	103†	21.2 (17.7–25.2)		57	2.9 (2.2–3.8)
Urinary system diseases and symptoms	102†	21.0 (17.5–24.9)		109	5.6 (4.6–6.8)
Gastrointestinal system diseases and symptoms	85†	17.5 (14.2–21.2)		122	6.3 (5.3–7.5)
Skin and soft tissue diseases	83†	17.1 (13.9–20.8)		82	4.2 (3.4–5.2)
Arthrosis and inflammatory polyarthropathies	59†	12.2 (9.4–15.4)		71	3.7 (2.9–4.6)
Metabolic disorders	51†	10.5 (7.9–13.6)		65	3.4 (2.6–4.3)
Infections of the skin and subcutaneous tissue	48†	9.9 (7.4–12.9)			
Diabetes mellitus	46†	9.5 (7.0–12.5)		67	3.5 (2.7–4.4)
Benign neoplasms	42†	8.7 (6.3–11.5)		55	2.8 (2.1–3.7)
Complications of surgical and medical care	38†	7.8 (5.6–10.6)		49	2.5 (1.9–3.3)
Circulatory and respiratory symptoms	37†	7.6 (5.4–10.4)		67	3.5 (2.7–4.4)
Diseases of veins, lymphatic vessels, and lymph nodes	31†	6.4 (4.4–9.0)			
Anemia	30†	6.2 (4.2–8.7)		NA	NA
Chronic lower respiratory diseases	30†	6.2 (4.2–8.7)		49	2.5 (1.9–3.3)
Other disorders of the ear	28§	5.8 (3.9–8.2)		89	4.6 (3.7–5.6)
Male genital organ diseases	28†	5.8 (3.9–8.2)		49	2.5 (1.9–3.3)
HIV	19†	3.9 (2.4–6.1)		4	0.2 (0.06−0.52)
Hematological malignancies	12¶	2.5 (1.3–4.3)		18	0.9 (0.6–1.5)

*NA, not available; NTM, nontuberculosis mycobacteria. †p<0.001. ‡Codes A30–A49 (excluding A31) in International Classification of Diseases, 8th Revision or 10th Revision. §Not significant. ¶p<0.01.

#### Break in Barrier Function

Several comorbidities related to break in barrier function were more common in extrapulmonary NTM patients than in controls. One third of extrapulmonary NTM patients (29.3%, 95% CI 25.3%–33.6%) had injuries to the head and musculoskeletal system, compared with 17.1% (95% CI 15.4–18.8) of controls. Complications from surgical and medical care were more common in extrapulmonary NTM patients: 7.8% (95% CI 5.6%–10.6%) from surgical care and 2.5% (95% CI 1.9%–3.3%) from medical care. Extrapulmonary NTM patients also experienced higher rates of skin and soft tissue diseases (17.1%, 95% CI 13.9%–20.8%) and diseases of veins and lymphatic tissues (6.4%, 95% CI 4.4%–9.0%). Extrapulmonary NTM patients had a higher proportion of urinary system disease at 21.0% (95% CI 17.5%–24.9%); of those, 8.8% (95% CI 4.1%–16.1%) had been treated with BCG intravesically before diagnosis.

#### Impaired Immunity

Pneumonia and other bacterial diseases were significantly more common in extrapulmonary NTM patients at 21.2% (95% CI 17.7%–25.2%) than among controls at 2.9% (95% CI 2.2%–3.8%). The same discrepancy applied to diabetes mellitus (9.5%, 95% CI 7.0%–12.5%), HIV (2.5%, 95% CI 1.3%–4.3%), and hematologic malignancies (2.5%, 95% CI 1.3%–4.3%). Last, the proportion of inflammatory polyarthropathies was higher in extrapulmonary NTM patients (12.2%, 95% CI 9.4%–15.4%) than in controls (3.7% 95% CI 2.9%–4.6%) (Table 4).

## Discussion

We present a comprehensive characterization of mortality rates among persons with extrapulmonary NTM infection and a unique description of causes of death in this nationwide register-based cohort study over an 18-year period. Patients with extrapulmonary NTM disease had higher all-cause mortality and died more often of hematologic malignancies and HIV than did controls.

Patients with extrapulmonary NTM infection had a higher mortality rate than controls in the first 3 years, and that difference persisted when controlling for comorbidities. Men and women with extrapulmonary NTM infection had similar all-cause mortality rates. The overall mortality rate was 32.6%, which is considerably higher than the rate of 4% reported in a recent Australia study of 73 patients with extrapulmonary NTM disease (*10*). That dissimilarity is likely because of differences in the 2 populations’ sample sizes, demographics, burden of comorbidities, and disease manifestations.

Previous studies have shown that mortality rates among patients with extrapulmonary NTM infection are lower than that associated with pulmonary NTM disease and that mortality varies by localization, patient population, and NTM species (*26*). Disseminated infections with *M. chimaera* from heater–cooler units have been associated with a case-fatality rate of 45.5% (*12*). Comparable high mortality rates have been reported in different extrapulmonary NTM studies on peritonitis in immunosuppressed persons, patients with central nervous system infection, and persons who have undergone renal or allogeneic stem cell transplant (21%–50%) (*27*–*30*).

Understanding the cause of death for persons with extrapulmonary NTM disease is essential to identify patients who could benefit from earlier intervention (*31*). In this study, extrapulmonary NTM patients most often died of an underlying comorbidity, and only 5.7% of deaths were attributable to other bacterial diseases, potentially including NTM disease. Deaths caused by hematologic malignancies and HIV were more common in persons with extrapulmonary infection, and those diseases are also considered risk factors for NTM disease (*2*,*10*). Still, when excluding persons with HIV from our analyses, the mortality rate remained higher among patients with extrapulmonary infection. We did not observe significant differences in the 2 most common causes of death for persons with extrapulmonary NTM disease and controls.

Persons with extrapulmonary NTM infection had a significantly higher burden of comorbidities than did controls. That finding was evident when investigating the CCI and common categories of comorbidities in the years before the diagnosis. The higher proportion of cardiovascular disease, metabolic disorders, and chronic lower respiratory disease in extrapulmonary NTM patients may indicate a higher degree of frailty, leading to an increased incidence of extrapulmonary NTM infection. Cardiovascular disease has previously been identified as a risk factor for pulmonary NTM disease but, to our knowledge, has not been identified as such for extrapulmonary infection (*25*).

Several comorbidities, which might provide a port of entry for mycobacteria because of compromised barrier function, were more common in patients with extrapulmonary NTM disease. Those patients experienced higher proportions of injuries; skin and subcutaneous tissue disease; and diseases of the veins, lymphatic vessels, and lymph nodes that all expose skin and soft tissues to risk for infection. Surgical and medical complications were also more common, indicating that extrapulmonary NTM infection can be seen as iatrogenic, which has been described consistently (*10*,*11*).

We observed a higher proportion of benign neoplasms before NTM disease was diagnosed. Invasive procedures in the diagnostic process or the neoplasms itself could lead to compromised barrier function. Neoplasms could also to some degree be misclassifications that are in fact granulomatous formation caused by NTM. Diseases of the urinary system and male genital organs were significantly more common in extrapulmonary NTM patients. Only a few of those patients (9/102) had intravesical BCG installation, which could have been a confounder, because BCG installations are known to cause BCGitis (*32*). NTM infections of the genitourinary tract are extremely rare and are unlikely to explain the large proportion of genitourinary diseases in our population (*33*). Disease in the genitourinary tract might indicate impaired mucosa barrier function or poor overall health, which increases the risk for extrapulmonary NTM. In addition, gastrointestinal diseases and symptoms were more prevalent in patients with extrapulmonary NTM infection, which could suggest compromised barrier function in the intestines. However, the extrapulmonary NTM disease could be localized to the gastrointestinal tract, as is the case with *M. genavense* (*34*).

Immunosuppression is strongly associated with extrapulmonary NTM (*35*,*36*). Immunosuppression in patients with any manifestation of NTM has been associated with an increased risk for death (HR 3.5 [95% CI 1.5–8.4]), but only 13 of 118 patients had an extrapulmonary NTM diagnosis (*37*). In our study, extrapulmonary NTM patients had a significantly higher proportion of hematological malignancies, which implies a higher risk for infection because of impaired immunity. Patients with extrapulmonary infection had higher proportions of anemia, HIV/AIDS, and diabetes mellitus, which are also associated with impaired immunity (*2*,*25*,*38*). Our study confirms an association with immunosuppression and HIV infection, which was also shown in an Australia study (*10*). Still, HIV generally seems to be rare in patients with NTM in our setting.

The higher proportion of inflammatory polyarthropathies in extrapulmonary NTM patients could be related to the use of immunosuppressants. Anti–tumor necrosis factor α treatment and corticosteroids are well-described risk factors for mycobacterial disease (*9*,*25*,*39*). A high mortality rate has been reported among patients treated with biological agents who have concomitant NTM infections (*9*).

The significantly higher proportion of extrapulmonary NTM patients with other bacterial diseases and pneumonia could be associated with an increased likelihood of immune dysfunction (*40*). Pneumonia is also a risk factor for pulmonary NTM (*25*). However, that finding could also be because of misdiagnoses and erroneous use of diagnostic codes.

This study was based on national registers, and the data entered in those registers imply an inherent risk for underreporting, overreporting, and misclassification. Treatment of extrapulmonary NTM disease is centralized in 6 hospitals in Denmark with specialized centers for mycobacterial diseases, and we are confident that the diagnoses have a high rate of accuracy. Previous validations of diagnostic codes in our registers have shown a high positive predictive value (*41*,*42*). The ICD-10 codes pertaining to extrapulmonary NTM manifestation and species are only specific for skin infections with *M. marinum* and *M. ulcerans*, and the registers used in this study do not contain data on microbiology or all relevant risk factors (e.g., type of immunosuppressants). This factor limited our ability to precisely describe the type, localization, and pathogenicity of NTM infections, including species. Nevertheless, the percentage of extrapulmonary NTM skin infections in our study (29.7%) is comparable to those in a US culture-based study (32%) (*16*). We assume that using ICD-10 codes for extrapulmonary NTM disease correlates well with clinically relevant disease compared with relying on microbiologic data alone. Furthermore, the inclusion of *M. ulcerans* patients could be debated, because *M. ulcerans* might not be considered as a separate disease entity, although it is genetically very similar to *M. marinum* (*6*,*43*,*44*).

Data on the causes of death are registered by the physician who verifies the death. However, those physicians might not be familiar with the patient’s entire medical history or with complex infections, making it difficult to differentiate between causes of death in this comorbid population. The low number of autopsies conducted in Denmark calls into question the validity of the death certificates; however, that issue appears to be common in other settings (*31*).

In conclusion, patients with extrapulmonary NTM infection have a significantly higher burden of comorbidities than do matched controls. The mortality rate remained higher for persons with extrapulmonary NTM infection than for matched controls after adjusting for a comorbidity severity index. In addition, patients with extrapulmonary NTM disease often die from underlying comorbidities, such as hematologic malignancies and HIV, not from extrapulmonary NTM disease.

Appendix Additional information about mortality rate and cause of death in adults with extrapulmonary nontuberculous mycobacteria infection, Denmark.
