# Supplementary material for: Mortality Rate and Cause of Death in Adults with Extrapulmonary Nontuberculous Mycobacteria Infection, Denmark
Source: Emerg Infect Dis. 2024 Sep;30(9):1790–8. doi: 10.3201/eid3009.240475 (PMC11346995; doi:10.3201/eid3009.240475)
Supplement: Appendix — Additional information about mortality rate and cause of death in adults with extrapulmonary nontuberculous mycobacteria infection, Denmark. [file 24-0475-Techapp-s1.pdf]

Article DOI: https://doi.org/10.3201/eid3009.240475

EID cannot ensure accessibility for supplementary materials supplied by authors. Readers who have difficulty accessing supplementary content should contact the authors for assistance.

Mortality Rate and Cause of Death in Adults with Extrapulmonary Nontuberculous Mycobacteria Infection, Denmark

Appendix

Appendix Table. Inclusion and exclusion criteria used to generate the extrapulmonary nontuberculous mycobacteria infection cohort.

Generation of extrapulmonary nontuberculous mycobacterial disease cohort
Inclusion criteria
Cutaneous mycobacterial infection (A31.1) OR<br>Cutaneous infection with *Mycobacterium marinum* (A31.1A) OR<br>Cutaneous infection with Mycobacterium ulcerans (A31.1B)
OR
Other mycobacterial infections/Unspecified (A31.8 & A31.9)
AND
No exclusion criteria
Exclusion criteria
Treatment with inhalation of colistin (BGHR9A) OR<br>Interventions due to secretions of the airways (BGME*) OR<br>Endoscopy of the trachea, bronchia and lungs (KUG*) OR<br>Gastric lavage (ZZ1010) OR<br>Surgery of the lung (KGD*) OR<br>Sputum collection for culturing (ZZ4172) OR<br>Bronchiectasis (J47.9) OR<br>Chronic obstructive pulmonary disease (J44.x) OR<br>Tuberculosis (A15, A16, B90)
